# Maternal parenting and the development of adolescent self‐esteem: A longitudinal twin study

**DOI:** 10.1111/jora.70298

**Published:** 2026-10-20

**Authors:** Jasmine Lim, Jiesi Guo, Christoph H. Klatzka, Ziwen Teuber

**Affiliations:** ^1^ Institute for Positive Psychology and Education Australian Catholic University North Sydney NSW Australia; ^2^ Department of Psychology Saarland University Saarbrücken Saarland Germany; ^3^ Department of Behavioural and Cognitive Sciences University of Luxembourg Esch‐sur‐Alzette Luxembourg

**Keywords:** adolescents, parenting, psychological control, self‐esteem, twin study, warmth

## Abstract

Adolescence is a critical period for self‐esteem development, yet the extent to which perceived maternal parenting practices are prospectively associated with self‐esteem after accounting for overlapping genetic and shared environmental influences remains unclear. Using longitudinal TwinLife data from 1524 German twins (323 monozygotic and 439 same‐sex dizygotic twin pairs), this study examined perceived maternal warmth and psychological control from ages 13 to 15. Classical twin models decomposed genetic and environmental variance and covariance, and ACE‐beta models estimated prospective associations with self‐esteem at age 15. Univariate ACE models indicated developmental shifts in the genetic and shared environmental contributions to perceived parenting, whereas self‐esteem showed a relatively stable pattern and substantial nonshared environmental variance. Bivariate ACE models showed a positive shared environmental correlation between warmth and self‐esteem (rC = .78) and a negative genetic correlation between psychological control and self‐esteem (rA = −.68). Within the ACE‐beta parameterization, warmth at age 13 was prospectively associated with higher self‐esteem at age 15 (β_21_ = .28), whereas psychological control was associated with lower self‐esteem (β_21_ = −.17), after adjustment for prior self‐esteem and covariates. These prospective associations are consistent with, but do not uniquely establish, directional effects of parenting on later self‐esteem. The findings support maternal warmth and autonomy‐respecting practices as plausible targets for promoting adolescent self‐worth.

## INTRODUCTION

Adolescence is a critical developmental period marked by significant transitions and psychosocial changes that shape individuals' sense of self and overall well‐being (Meschke et al., 2012). Among the contextual factors associated with psychological adjustment during this period, parenting is consistently related to adolescents' self‐esteem (Pinquart, 2023). Although both constructs have been studied extensively, less is known about their prospective association after genetic and environmental influences are considered. Using a classical twin design comparing monozygotic and dizygotic twins from ages 13 to 15, this study examines associations between perceived maternal warmth and psychological control and later self‐esteem after accounting for covariance allocated to overlapping genetic and shared environmental pathways and prior self‐esteem. This approach provides a genetically informed account of how perceived parenting and self‐esteem are related across adolescence and may inform tailored interventions during this formative developmental stage.

### Self‐esteem

Self‐esteem, defined as an individual's positive or negative attitude toward themselves (Rosenberg, 1965), is fundamental to psychological health and well‐being. A framework for understanding the development of self‐esteem is self‐determination theory (SDT; Deci & Ryan, 1985), which posits that all human beings have the fundamental needs to feel related, competent, and autonomous to develop and function optimally (Ryan & Deci, 2000). When adolescents perceive their social environment as supportive of these needs, they are more likely to develop stable and positive self‐esteem, whereas environments that thwart these needs undermine self‐worth and adjustment (Ryan & Deci, 2000).

Self‐esteem has traditionally been examined as an outcome of environmental influences, with socialization experiences, peer interactions, and broader contextual factors shaping how individuals evaluate themselves (Bandura, 1977; Baumrind, 1971). However, behavioral‐genetic research shows that self‐esteem is also moderately heritable (Neiss et al., 2002). Behavioral‐genetic studies typically estimate that genetic differences account for approximately 30% to 50% of individual differences in self‐esteem (Kendler et al., 1998; Neiss et al., 2006). In twin studies, variance is commonly decomposed into additive genetic effects, shared environmental effects, and nonshared environmental effects, the last of which also include measurement error.

Longitudinal twin evidence indicates that the relative contributions of genetic and environmental influences to self‐esteem can vary across development. Jonassaint (2010) analyzed 289 monozygotic and 452 dizygotic twin pairs from the U.S. National Longitudinal Study of Adolescent Health. Participants were 10–20 years old at baseline, with mean ages of approximately 14.9, 16.5, and 21.8 years across the three assessments. Additive genetic influences accounted for approximately 43%, 45%, and 13% of self‐esteem variance, respectively; the remaining variance was attributed principally to nonshared environmental influences (approximately 57%, 55%, and 87%).

In a population‐based Finnish twin cohort, Raevuori et al. (2007) assessed 4132 adolescents at age 14 and 3841 at age 17. For boys, additive genetic and nonshared environmental estimates were .62 and .38 at age 14 and .48 and .52 at age 17. For girls, additive genetic, shared environmental, and nonshared environmental estimates were .40, .31, and .30 at age 14 and .29, .34, and .37 at age 17. These findings support developmental variation but do not indicate a uniform linear decline in heritability across adolescence.

### Perceived parenting practices

Parenting practices have long been recognized as important correlates of children's psychological adjustment and well‐being (Harter, 2015). The present study focuses on two dimensions of perceived maternal parenting: warmth and psychological control. Warmth reflects expressions of affection, support, and acceptance, whereas psychological control involves manipulative or intrusive practices that restrict autonomy and self‐expression (Barber et al., 2005; Bradshaw et al., 2025). Adolescents who perceive their parents as warm, responsive, and supportive tend to report better psychological adjustment, whereas those who perceive controlling or rejecting parenting tend to report lower self‐esteem (Baumrind, 1966; Steinberg, 2001).

Although parenting is often conceptualized as an environmental influence, twin and family studies indicate that adolescents' perceptions of parenting are partly heritable (Hur & Bouchard, 1995). For instance, Kendler and Baker (2007) found that children's perceptions of parental behaviors were heritable, with higher heritability for parental warmth (35%) than for other dimensions of parental behavior (19%–23%). Meta‐analytic child‐based estimates suggest that additive genetic influences account for approximately 26% of variance in perceived parental warmth and 23% in parental control, whereas nonshared environmental influences account for approximately 34% and 44%, respectively (Klahr & Burt, 2014). Thus, compared with parental warmth, parental control appears somewhat less heritable and more strongly influenced by nonshared environmental factors.

Genetically influenced child characteristics, such as temperament or sensitivity to social feedback, may shape how parenting behaviors are experienced and interpreted. Part of this heritability may also reflect parental genetic influences, whereby mothers' genetically influenced characteristics affect their parenting behaviors and are transmitted to children through shared genetics. Evidence for genetic influence on perceived parenting is not limited to adolescent reports. In an adult twin sample, Marshall et al. (2021) found that retrospective reports of maternal and paternal affection were significantly heritable, indicating that remembered parenting experiences may also reflect genetically influenced individual differences in perception and recall.

In child‐based twin models, the nonshared environmental component (E) captures experiences and perceptions that differentiate co‐twins, together with measurement error. A larger E estimate therefore indicates a larger proportion of variance not shared by co‐twins; it, however, does not identify the source of that variance or establish a causal environmental effect. Genetically informed designs are consequently useful for examining whether covariance between perceived parental control and outcomes such as self‐esteem is allocated to genetic, shared environmental, or nonshared environmental components.

Beyond these overall genetic and environmental contributions, longitudinal evidence suggests that the genetic and environmental processes underlying perceived parenting may change across development. Hannigan et al. (2017) examined children's reports of harsh parental discipline from ages 9 to 16 and negative parental feelings from ages 9 to 12. These constructs are conceptually adjacent to, rather than direct measures of, psychological control and warmth; the negative parental feelings measure is more closely aligned with low warmth. The findings did not indicate a simple decline in heritability across adolescence. Instead, newly emerging, age‐specific genetic and nonshared environmental influences contributed to change in perceived parenting, whereas shared environmental influences contributed more to stability. These findings suggest that the etiological processes underlying perceived parenting are developmentally dynamic and highlight the value of longitudinal, genetically informed research on maternal warmth and psychological control.

### The relationship between maternal parenting and self‐esteem

A substantial body of research has established that maternal parenting plays a crucial role in shaping adolescents' self‐esteem. Mothers often serve as primary attachment figures during childhood and adolescence, and maternal warmth, responsiveness, and autonomy support provide a foundational relational context in which adolescents develop a sense of worth and belonging (Belsky, 1984; Maccoby, 1984). Consistent with this perspective, maternal warmth and support are reliably associated with higher self‐esteem, whereas maternal psychological control or rejection is linked to lower self‐esteem and poorer adjustment (Pinquart & Gerke, 2019).

Despite the consistency of these associations, it remains unclear whether the link between perceived maternal parenting and self‐esteem reflects a directional influence of parenting, overlapping genetic or environmental influences, or both. Both perceived parenting and self‐esteem are partly heritable, so their association may arise partly from common genetic or environmental factors rather than a unidirectional effect of parenting (Kendler & Baker, 2007). For example, adolescents with heritable dispositions toward positive affect, sociability, or emotional stability may be more likely to perceive their mothers as warm and supportive while holding more positive self‐evaluations. Such processes can generate phenotypic associations without requiring a direct environmental effect of parenting (Patterson et al., 2017; South et al., 2008). This pattern reflects gene–environment correlation (rGE), whereby genetically influenced characteristics shape the environments individuals experience (Scarr & McCartney, 1983).

Empirical findings suggest that these genetically influenced processes may be particularly pronounced in maternal parenting. For instance, maternal warmth has been shown to correlate with offspring self‐esteem through shared genetic factors (Marshall et al., 2021). Comparative studies also indicate that mothers may be more responsive than fathers to child‐driven genetic characteristics, especially in domains involving autonomy regulation and psychological control (Neiderhiser et al., 2004, 2007). Meta‐analytic evidence further indicates stronger child‐genetic influence on maternal control and negativity than on corresponding paternal behaviors (Klahr & Burt, 2014). These findings highlight the value of genetically informative designs for examining how genetic, shared environmental, and nonshared environmental processes contribute to associations between maternal parenting and self‐esteem.

### The present study

To address these gaps, the present study uses a longitudinal, genetically informed twin design to examine prospective associations between perceived maternal warmth and psychological control at age 13 and self‐esteem at age 15. The twin design partitions covariance into genetic, shared environmental, and nonshared environmental components, allowing us to estimate the prospective association between perceived maternal parenting and later self‐esteem after accounting for covariance allocated to overlapping genetic and shared environmental pathways. We also adjust for prior self‐esteem. By clarifying these pathways and the identifying assumptions of the ACE‐beta parameterization, this study aims to advance understanding of how perceived maternal parenting and adolescents' self‐esteem are related across early to mid‐adolescence.

The study aims to address the following questions:

1. What proportion of variance in perceived maternal parenting and self‐esteem is attributable to genetic, shared environmental, and nonshared environmental factors at ages 13 and 15?
2. To what extent do perceived maternal parenting and self‐esteem share genetic, shared environmental, or nonshared environmental influences across ages 13 and 15?
3. To what extent are perceived maternal warmth and psychological control at age 13 prospectively associated with self‐esteem at age 15 after accounting for covariance attributable to overlapping genetic and shared environmental pathways and prior self‐esteem?

## METHODS

### Sample

Data for this project were drawn from the TwinLife project (Diewald et al., 2025), a cross‐sequential study of more than 4000 German twin families designed to investigate social inequality across the lifespan. The present study used data from Cohort 2, comprising twins born in 2003/2004, with assessments from two face‐to‐face waves: Wave 3, referred to as T1 (*n* = 1496 individual twins), and Wave 5, referred to as T2 (*n* = 1271 individual twins). A total of 296 adolescents who participated at Wave 3 did not participate at Wave 5, whereas 71 adolescents re‐entered the study at Wave 5 after participating in earlier waves but not Wave 3.

The T1 and T2 sample sizes reported above refer to individual twins, not twin pairs. The final analytic sample represents unique individual twins retained across Wave 3 and Wave 5 after family‐level, zygosity, and construct‐availability criteria were applied. Twin pairs were retained when both twins had valid zygosity information and complete data on maternal warmth, psychological control, and self‐esteem in at least one of the two waves. Because participation varied across waves, the analytic sample was not restricted to complete cases at both T1 and T2. The final longitudinal analytic sample comprised 1524 unique individual twins. Of these, 1463 participated at T1 and 1255 at T2, with 1194 assessed at both waves. The sample included 323 monozygotic (MZ) and 439 same‐sex dizygotic (DZ) twin pairs and was approximately evenly distributed by sex (T1: 50% female; T2: 51% female).

### Procedures

The present study used face‐to‐face TwinLife assessments. In these survey waves, trained interviewers visited participating families in their homes. Data were collected using multiple modes, including computer‐assisted personal interviews, computer‐assisted self‐interviews on tablet computers, and paper‐and‐pencil questionnaires. Multiple devices allowed family members to complete portions of the household assessment concurrently. Respondents received compensation for participating in the TwinLife surveys. The parenting measures were completed electronically, whereas self‐esteem was completed using a paper‐and‐pencil questionnaire. Participants could move to another room if they wished, and interviewers were instructed to ensure that other household members did not intervene while participants completed the questionnaires. Although physical separation from parents and the co‐twin was not required, these procedures were intended to support independent responding.

### Measures

#### Perceived maternal parenting practices

Perceived maternal warmth was assessed with four adolescent‐report items from a 13‐item questionnaire adapted from pairfam (Huinink et al., 2011; e.g., “Your mother praises you”). Perceived maternal control was assessed using the three‐item composite labeled “Psychological Control” in the TwinLife documentation. The items were as follows: “Your mother punishes you when you do something against her will”; “Your mother is disappointed and sad because you misbehaved”; and “Your mother makes it clear to you that you are not to break the rules or question her decisions.” The items span punitive discipline, emotion‐based disapproval, and restriction on questioning parental authority. Accordingly, the composite should be interpreted as a broad, heterogeneous indicator of perceived maternal control rather than a narrowly defined measure of psychological control. Participants responded on a scale from 1 (“Never”) to 5 (“Very Often”). Internal consistency was good for warmth (Cronbach's α = .84 at T1 and .78 at T2) and acceptable for psychological control (α = .72 at T1 and .67 at T2).

#### Self‐esteem

Self‐esteem was assessed using a three‐item brief version of the Rosenberg Self‐Esteem Scale (Rosenberg, 1965) documented in the pairfam scales manual (Thoennissen et al., 2015). Participants completed the items (“On the whole, I am satisfied with myself”, “At times I think that I am no good at all” (reverse scored), “I take a positive attitude towards myself”) using a Likert scale ranging from 1 (“Strongly disagree”) to 5 (“Strongly agree”). Internal consistency was acceptable at both time points, with Cronbach's α = .75 (T1) and .76 (T2).

#### Covariates

Family Household Composition (FHC) refers to the number of households living within a single dwelling, with higher values indicating more co‐residing households (e.g., 1 = one household, 2 = two households, and 3 = three households). A greater number of households within one dwelling was treated as an indicator of lower socioeconomic status due to housing constraints or financial limitations. Sex was coded 1 = male and 2 = female.

### Analytic plan

The present study used five analytic approaches: (1) longitudinal measurement invariance testing, (2) descriptive analyses, (3) univariate variance decomposition, (4) bivariate genetic and environmental correlations, and (5) bivariate ACE‐beta models with covariates. All analyses were conducted in R (R Core Team, 2024). Structural equation models were estimated using the lavaan package, and genetic analyses were conducted using the umx package (Bates et al., 2019) and twinflex (Ruks, 2022), a wrapper for OpenMx (Neale et al., 2016). Missing data were handled using full information maximum likelihood (FIML). Analysis scripts and annotated R code are publicly available through the Open Science Framework (OSF; https://osf.io/8fqs5/overview?view_only=29e36c41db454971b818854c87b1429c).

#### Longitudinal measurement invariance

Before twin modeling, longitudinal measurement invariance of the parenting and self‐esteem measures was tested across ages 13 and 15 using confirmatory factor analysis in lavaan. Model fit was evaluated using the comparative fit index (CFI), Tucker–Lewis index (TLI), and root‐mean‐square error of approximation (RMSEA). Values of CFI and TLI of .90 and .95 indicate acceptable and excellent fit, respectively, whereas RMSEA values of .08 and .05 indicate acceptable and close fit. Hu and Bentler (1999) recommended the more stringent approximate values of .95 for CFI/TLI and .06 for RMSEA in combination‐rule evaluations. Configural, metric, and scalar invariance were tested sequentially. Changes of no more than .01 in CFI and TLI and no more than .015 in RMSEA were taken as support for the more constrained model (Chen, 2007; Cheung & Rensvold, 2002).

Standardized factor scores derived from the scalar‐invariance models were used in the twin analyses. Relative to raw summed scores, factor scores may reduce item‐specific unreliability that would otherwise contribute to the nonshared environmental component E. Nevertheless, factor scores remain fallible estimates and were treated as observed variables in the ACE models; consequently, residual factor‐score estimation error may still contribute to E.

#### Descriptive analyses

Descriptive analyses were conducted to examine sample characteristics, mean scores, and standard deviations for perceived parenting and self‐esteem at both time points. Zero‐order phenotypic correlations were calculated to examine bivariate associations among variables. Twin correlations were also computed separately for MZ and DZ twin pairs to provide initial evidence of genetic and environmental influences.

#### Univariate ACE models

Univariate ACE models (see Figure 1a) were used to decompose variance in perceived maternal parenting and self‐esteem into additive genetic (A), shared environmental (C), and nonshared environmental (E) components. Nonadditive genetic effects (D) were also considered when indicated by the twin correlations. Shared environmental effects (C) and nonadditive genetic effects (D) imply different expected covariance patterns for DZ twins. However, the classical twin design based only on MZ and DZ twins reared together does not provide sufficient degrees of freedom to estimate A, C, D, and E simultaneously. Accordingly, C and D were not included in the same model; ACE or ADE models were fitted according to the observed MZ and DZ twin‐correlation patterns (Knopik et al., 2017; Neale & Cardon, 2013). Nonshared environmental estimates include influences that differentiate co‐twins as well as measurement error.

**FIGURE 1:**
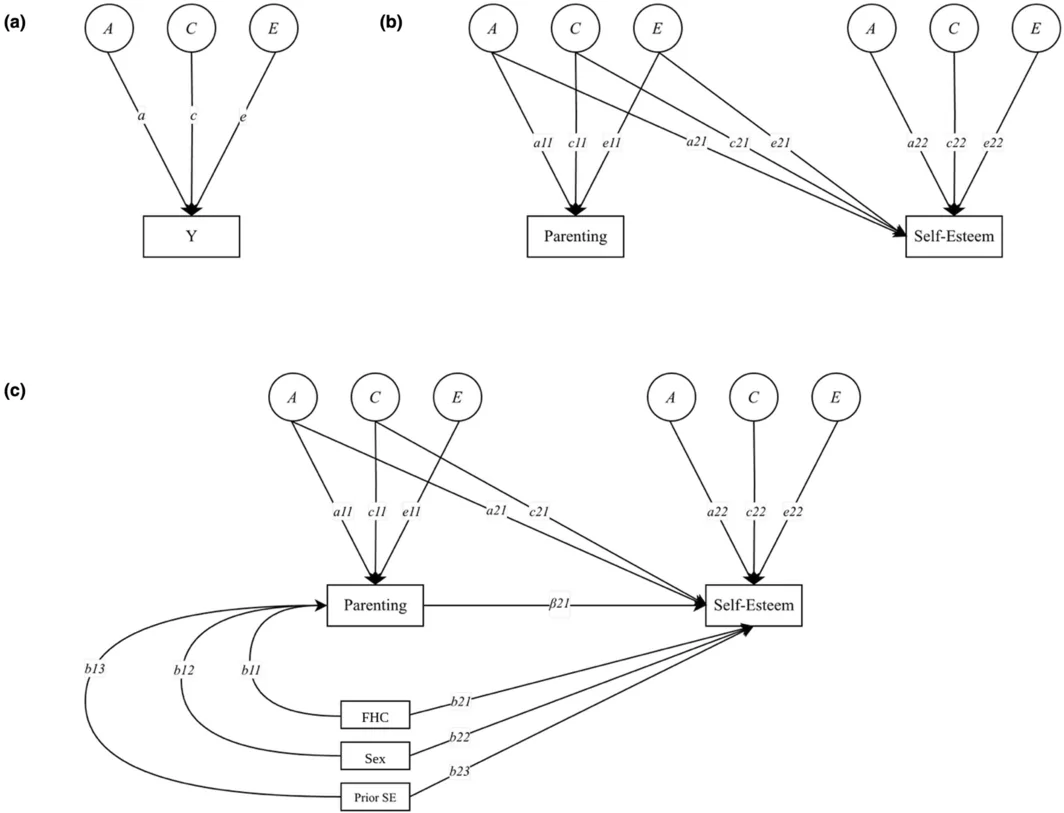
Path diagrams of estimated twin models. A, Univariate ACE model; B, Bivariate ACE (Cholesky decomposition) model; C, Bivariate ACE‐beta model.

#### Bivariate **ACE** models

Bivariate ACE models (see Figure 1b) were estimated to decompose the covariance between each perceived parenting practice at age 13 and self‐esteem at age 15 into overlapping genetic, shared environmental, and nonshared environmental components. Genetic (rA), shared environmental (rC), and nonshared environmental (rE) correlations were calculated. For example, rA represents the extent to which additive genetic factors influencing perceived parenting overlap with those influencing later self‐esteem.

Confidence intervals for genetic, shared environmental, and nonshared environmental correlations were obtained using a nonparametric bootstrap with 1000 replications per model. MZ and DZ twin‐pair rows were resampled with replacement within their respective zygosity data sets; each bivariate model was refitted, and the derived correlations were recalculated in each replication. Percentile 95% confidence intervals are reported.

The ACE‐beta parameterization (see Figure 1c) replaces the cross‐trait nonshared environmental path (e_21_) with a phenotypic regression path (β_21_). With only MZ and DZ twins reared together, e_21_ and β_21_ cannot be freely estimated simultaneously. The bivariate ACE model therefore estimates e_21_ while fixing β_21_ = 0, whereas the ACE‐beta model estimates β_21_ while fixing e_21_ = 0. These parameterizations reproduce the same observed covariance structure under different identifying constraints, so model fit cannot distinguish a directional pathway from common nonshared environmental covariance. The beta coefficient quantifies the prospective pathway conditional on e_21_ = 0; it is therefore informative about the temporally ordered association but does not uniquely identify a directional effect. FHC, sex, and prior self‐esteem at age 13 were subsequently added as covariates.

Maternal warmth and psychological control were modeled separately in the bivariate ACE and ACE‐beta analyses. This decision was made to preserve model parsimony, estimation stability, and interpretability. Simultaneously decomposing multiple predictors into genetic, shared environmental, and nonshared environmental components would substantially increase the number of estimated paths and covariance parameters, which can reduce estimation precision and increase the risk of identification or convergence problems in multivariate behavioral‐genetic models (Knopik et al., 2017; Verhulst et al., 2019). The two parenting dimensions were related but distinct in the present sample, as indicated by their moderate negative phenotypic correlation at age 13 (*r* = −.33), supporting separate focal tests of the longitudinal association between each parenting dimension and subsequent self‐esteem.

## RESULTS

### Longitudinal measurement invariance

Longitudinal measurement invariance was examined using confirmatory factor analysis, with warmth, psychological control, and self‐esteem modeled simultaneously across time. The configural model demonstrated acceptable fit, χ^2^(155) = 710.61, *p* < .001, CFI = .94, TLI = .93, RMSEA = .048. The metric model also showed acceptable fit, χ^2^(162) = 738.15, *p* < .001, CFI = .94, TLI = .93, RMSEA = .048, with minimal changes in fit (ΔCFI = −.001, ΔTLI = .001, ΔRMSEA = .001). The scalar model maintained acceptable fit, χ^2^(169) = 777.81, *p* < .001, CFI = .94, TLI = .93, RMSEA = .049, with minimal additional change (ΔCFI = −.003, ΔTLI = −.001, ΔRMSEA = .001). These results supported scalar invariance, and standardized factor scores were extracted for the subsequent twin analyses.

### Descriptives

Table 1 presents the sample characteristics and descriptive statistics for the primary study variables. Table 2 reports zero‐order correlations among perceived maternal parenting dimensions and adolescent self‐esteem at ages 13 and 15. These results indicate that maternal warmth is consistently associated with higher adolescent self‐esteem, whereas maternal psychological control shows small negative associations. Both warmth and psychological control demonstrated moderate stability over time and substantial correlations.

**TABLE 1:** Sample characteristics, mean scores, and standard deviations.

	T1 (*N* = 1463)		T2 (*N* = 1255)	
	*M*	SD	*M*	SD
Age	13.02	0.33	15.07	0.34
Warmth	4.28	0.60	4.20	0.63
Psychological control	2.95	0.78	3.02	0.78
Self‐esteem	4.18	0.77	4.03	0.86
Sex (Female, %)	50	‐	51	‐

Abbreviations: *M*, mean; SD, standard deviation; T1, Time 1 (age 13); T2, Time 2 (age 15).

**TABLE 2:** Zero‐order correlations among perceived maternal parenting and self‐esteem.

Variable	1	2	3	4	5	6	7	8
Age 13								
1. Warmth	‐							
2. Psychological Control	−.33**	‐						
3. Self‐Esteem	.44**	−.23**	‐					
Age 15								
4. Warmth	.74**	−.26**	.30**	‐				
5. Psychological Control	−.34**	.85**	−.17**	−.29**	‐			
6. Self‐Esteem	.30**	−.14**	.61**	.40**	−.10**	‐		
Covariates								
7. Sex	.06*	−.21**	−.14**	.04	−.20**	−.24**	‐	
8. FHC	−.06*	.02	−.06*	−.07**	.02	−.06*	.02	‐

*Note*: Values represent Pearson's r. *p < .05. **p < .01. The correlation matrix was calculated from factor scores for the full sample (*N* = 1524).

Table 3 reports the twin correlations. Across all measures, MZ correlations (.43–.57) exceeded DZ correlations (.29–.44), indicating genetic influence. MZ correlations were less than twice the corresponding DZ correlations, and DZ correlations were greater than half the MZ correlations, a pattern consistent with additive genetic and shared environmental effects. ACE models were therefore retained for the subsequent analyses.

**TABLE 3:** Twin correlations.

Variable	Twin correlations			
	Age 13		Age 15	
	MZ (*n* = 312)	DZ (*n* = 419)	MZ (*n* = 273)	DZ (*n* = 353)
Warmth	.57***	.31***	.57***	.44***
Psychological Control	.52***	.40***	.57***	.40***
Self‐Esteem	.43***	.29***	.45***	.34***

*Note*: *n* denotes the number of twin pairs included in each wave‐specific intraclass correlation (pair‐complete participation counts). ***p < .001.

Abbreviations: DZ, dizygotic; MZ, monozygotic.

### Univariate genetic analyses

The results of the univariate ACE models are presented in Table 4. At age 13, the estimated proportions of variance attributable to A, C, and E were .41 [.20, .62], .13 [.03, .22], and .46 [.39, .53] for warmth; .14 [.03, .25], .36 [.18, .50], and .50 [.43, .58] for psychological control; and .22 [.04, .41], .19 [.05, .33], and .59 [.50, .67] for self‐esteem. At age 15, the corresponding estimates were .13 [.02, .24], .40 [.25, .56], and .46 [.40, .54] for warmth; .31 [.12, .51], .25 [.08, .41], and .44 [.37, .51] for psychological control; and .24 [.06, .41], .22 [.04, .40], and .55 [.46, .63] for self‐esteem. Bracketed values are 95% confidence intervals.

**TABLE 4:** Results of univariate models.

Variable	*a* ^*2*^	*c* ^*2*^	*e* ^*2*^
Age 13			
Warmth	.41 [.20, .62]	.13 [.03, .22]	.46 [.39, .53]
Psychological control	.14 [.03, .25]	.36 [.18, .50]	.50 [.43, .58]
Self‐esteem	.22 [.04, .41]	.19 [.05, .33]	.59 [.50, .67]
Age 15			
Warmth	.13 [.02, .24]	.40 [.25, .56]	.46 [.40, .54]
Psychological control	.31 [.12, .51]	.25 [.08, .41]	.44 [.37, .51]
Self‐esteem	.24 [.06, .41]	.22 [.04, .40]	.55 [.46, .63]

*Note*: Values are standardized proportions of variance; values in brackets are 95% confidence intervals.

Abbreviations: *a*2, additive genetic variance; *c* ^2^, shared environmental variance; *e* ^2^, nonshared environmental variance.

These estimates indicate a changing developmental pattern in the relative contributions of genetic and environmental variance. For warmth, the estimated genetic contribution was lower and the shared environmental contribution higher at age 15 than at age 13. Psychological control showed the reverse descriptive pattern, with a higher genetic estimate and a lower shared environmental estimate at age 15.

Self‐esteem showed a comparatively stable etiological pattern across ages 13 and 15. The nonshared environmental component was the largest estimated source of variance at both ages, although this component includes residual measurement and factor‐score estimation error and should not be attributed entirely to substantive individual‐specific experiences.

### Bivariate genetic analyses

#### Bivariate ACE models

##### Relationship between maternal warmth and self‐esteem

Maternal warmth and self‐esteem showed a positive shared environmental correlation (rC = .78, 95% CI [.22, 1.00]), although the interval was wide. The genetic correlation was modest, and its interval included zero (rA = .20, 95% CI [−.33, .73]). The nonshared environmental correlation was positive and comparatively precise (rE = .22, 95% CI [.14, .31]).

##### Relationship between maternal psychological control and self‐esteem

Psychological control and self‐esteem showed a negative genetic correlation (rA = −.68, 95% CI [−1.00, −.08]). The shared environmental correlation was small, and its interval included zero (rC = .15, 95% CI [−.17, .66]), whereas the nonshared environmental correlation was small and negative (rE = −.11, 95% CI [−.20, −.04]).

#### Bivariate ACE‐Beta models

##### Prospective association of maternal warmth with self‐esteem

Within the ACE‐beta parameterization, maternal warmth at age 13 was prospectively associated with self‐esteem at age 15 (β_21_ = .24, *p* < .001; Model 2, Table 5). The estimate was unchanged after adjustment for FHC and sex (β_21_ = .24, *p* < .001; Model 3) and increased after further adjustment for age‐13 self‐esteem (β_21_ = .28, *p* < .001; Model 4). The estimated prospective β_21_ path therefore remained after accounting for covariance allocated to overlapping genetic and shared environmental pathways in the fitted model and for measured prior self‐esteem.

**TABLE 5:** Results of best‐fitting bivariate models for perceived maternal warmth and self‐esteem.

	Model 1		Model 2		Model 3		Model 4	
	Bivariate ACE model		Bivariate ACE‐Beta model		Covariates model (FHC, sex)		Covariates model (FHC, sex, prior self‐esteem)	
	Estimate	SE	Estimate	SE	Estimate	SE	Estimate	SE
Warmth and self‐esteem								
a11	.64***	0.08	.64***	0.08	.63***	0.08	.56***	0.09
c11	.34**	0.13	.34**	0.13	.35**	0.13	.16	0.24
e11	.67***	0.02	.67***	0.02	.68***	0.03	.69***	0.03
a22	.48***	0.11	.48***	0.11	.50***	0.10	.34***	0.05
c22	.29	0.25	.29	0.25	.08	0.83	.00	0.18
e22	.72***	0.03	.72***	0.03	.71***	0.03	.73***	0.02
Warmth → self‐esteem								
a21	.10	0.12	−.06	0.14	−.02	0.14	−.20*	0.09
c21	.36	0.20	.28	0.21	.27	0.19	−.05	0.16
e_21_	.16***	0.04	‐		‐		‐	
β21	‐		.24***	0.06	.24***	0.06	.28***	0.05
FHC								
b11 (Warmth)	‐		‐		−.05	0.04	−.01	0.03
b21 (Self‐Esteem)	‐		‐		−.07	0.04	−.03	0.03
Sex								
b12 (Warmth)	‐		‐		.06*	0.03	.13***	0.03
b22 (Self‐Esteem)	‐		‐		−.25***	0.03	−.18***	0.02
Self‐Esteem (Age 13)								
b13 (Warmth)	‐		‐		‐		.25***	0.02
b23 (Self‐Esteem)	‐		‐		‐		.24***	0.02
AIC/BIC	8220.84/8236.55		8220.84/8236.55		8132.20/8153.46		7643.92/7667.93	
Δ‐2LL	‐		‐		96.93*** (vs. Model 2)		492.46*** (vs. Model 3)	

*Note*: Standardized path coefficients from bivariate ACE and ACE‐beta models predicting age‐15 self‐esteem from maternal warmth at age 13. *p < .05. **p < .01. ***p < .001.

Abbreviation: FHC, family household composition; SE, standard error.

##### Prospective association of maternal psychological control with self‐esteem

Within the ACE‐beta parameterization, psychological control at age 13 was prospectively associated with lower self‐esteem at age 15 (β_21_ = −.12, *p* < .05; Model 2, Table 6). The estimate remained −.12 after adjustment for FHC and sex (*p* < .05; Model 3) and increased in absolute magnitude after further adjustment for age‐13 self‐esteem (β_21_ = −.17, *p* < .01; Model 4).

**TABLE 6:** Results of best‐fitting bivariate models for perceived maternal psychological control and self‐esteem.

	Model 1		Model 2		Model 3		Model 4	
	Bivariate ACE model		Bivariate ACE‐Beta model		Covariates model (FHC, sex)		Covariates model (FHC, sex, prior self‐esteem)	
	Estimate	SE	Estimate	SE	Estimate	SE	Estimate	SE
Psychological control and self‐esteem								
a11	.37**	0.14	.37**	0.14	.36*	0.14	.27	0.18
c11	.60***	0.07	.60***	0.07	.57***	0.08	.55***	0.07
e11	.71***	0.03	.71***	0.03	.71***	0.03	.72***	0.03
a22	.36	0.23	.36	0.23	.43*	0.18	.33***	0.07
c22	.47***	0.10	.47***	0.10	.35**	0.13	.00	0.18
e22	.73***	0.03	.73***	0.03	.72***	0.03	.74***	0.02
Psychological control → self‐esteem								
a21	−.33	0.22	−.29	0.24	−.26	0.24	.00	0.24
c21	.07	0.11	.14	0.10	.03	0.10	.14	0.08
e_21_	−.08*	0.04	‐		‐		‐	
β21	‐	‐	−.12*	0.05	−.12*	0.05	−.17**	0.05
FHC								
b11 (Psychological Control)	‐		‐		.01	0.04	−.01	0.04
b21 (Self‐Esteem)	‐		‐		−.08*	0.04	−.03	0.03
Sex								
b12 (Psychological Control)	‐		‐		−.21***	0.03	−.25***	0.03
b22 (Self‐Esteem)	‐		‐		−.26***	0.03	−.19***	0.03
Self‐esteem (Age 13)								
b13 (Psychological Control)	‐		‐		‐		−.15***	0.02
b23 (Self‐Esteem)	‐		‐		‐		.29***	0.02
AIC/BIC	8318.49/8334.20		8318.49/8334.20		8187.68/8208.94		7772.73/7796.73	
Δ‐2LL			‐		139.10*** (vs. Model 2)		419.13*** (vs. Model 3)	

*Note*: Standardized coefficients from bivariate ACE and ACE‐beta models predicting age‐15 self‐esteem from age‐13 psychological control. * *p* < .05. **p < .01. ***p < .001.

Abbreviation: FHC, family household composition; SE, standard error.

These coefficients are estimated under the constraint e_21_ = 0. The bivariate ACE models also produced nonzero cross‐trait nonshared environmental correlations, showing that the associations can alternatively be represented through common nonshared influences. The beta coefficients are therefore interpreted as conditional prospective associations rather than uniquely identified directional effects.

## DISCUSSION

The present study used a longitudinal twin design to examine genetic and environmental contributions to perceived maternal parenting and adolescent self‐esteem and to estimate their prospective associations from early to mid‐adolescence. The findings show developmental variation in the variance components and distinct patterns of genetic, shared environmental, and nonshared environmental overlap between maternal warmth, psychological control, and self‐esteem. The ACE‐beta analyses further quantify temporally ordered associations under an explicit identifying constraint, but they do not uniquely identify causal effects.

### Sources of variance in parenting and self‐esteem

Consistent with prior behavioral‐genetic findings (Klahr & Burt, 2014; Neiss et al., 2002), genetic and environmental processes contributed to adolescents' perceptions of parenting and to self‐esteem, although their estimated magnitudes differed across constructs and ages. At age 13, maternal warmth was moderately heritable, suggesting that adolescents' genetically influenced dispositions, such as sociability, emotionality, or sensitivity to social feedback, may partly shape how they experience or elicit maternal warmth. By age 15, however, the shared environmental estimate was substantially larger, indicating a greater contribution of factors that make co‐twins similar. These factors may include aspects of the broader family context, such as emotional climate, cohesion, and shared parent–child experiences. Consistent with family‐systems and SDT perspectives (Bowen, 1978; Ryan & Deci, 2000), such shared contextual influences may become increasingly salient as adolescents mature and family relationships adapt to their growing autonomy.

Psychological control showed the opposite descriptive pattern: the genetic estimate was higher and the shared environmental estimate lower at age 15 than at age 13. One possible explanation is that adolescents' genetically influenced characteristics increasingly shape their perceptions of, or evoke, controlling parenting responses. Such evocative gene–environment processes are consistent with prior twin research (Klahr et al., 2013; Reiss et al., 2022), although the present variance decomposition cannot distinguish perception‐based from behavior‐based mechanisms.

Self‐esteem showed a relatively stable etiological pattern from ages 13 to 15, with modest genetic and shared environmental estimates and a larger nonshared environmental estimate. Genetic influences may partly reflect self‐evaluative tendencies associated with temperament or emotional stability, whereas shared environmental influences may include broad contextual factors that make co‐twins similar (Neiss et al., 2002). The nonshared environmental component captures processes that differentiate co‐twins, including individual‐specific experiences and perceptions, but it also includes residual measurement and factor‐score estimation error. It should therefore not be interpreted as a pure measure of substantive individual‐specific environmental influence. Nevertheless, the relatively large nonshared environmental component indicates substantial variation in self‐esteem that is not shared by co‐twins. Such differences may arise from individual‐specific experiences or from differences in how siblings experience and interpret similar social and family contexts (Plomin et al., 2001).

### Genetic and environmental overlap between parenting and self‐esteem

The findings revealed distinct patterns across parenting dimensions, highlighting that warmth and psychological control are linked to self‐esteem through different underlying mechanisms. For maternal warmth, the association with self‐esteem was primarily explained by shared environmental factors, with a strong positive shared environmental correlation. This suggests that family‐wide conditions promoting warmth, such as emotional cohesion, open communication, and supportive parent–child relationships, also foster higher self‐esteem in adolescents. Such shared environmental processes likely reflect a broader family emotional climate that nurtures adolescents' sense of competence and belonging, consistent with family‐systems and SDT perspectives (Bowen, 1978; Ryan & Deci, 2000). By contrast, psychological control exhibited a different pattern with a strong negative genetic correlation. It indicates that the association between psychological control and self‐esteem is predominantly driven by common genetic factors rather than by direct environmental transmission. In other words, the same heritable traits that predispose adolescents to perceive greater parental control (e.g., irritability or low self‐worth) also confer vulnerability to lower self‐esteem. This pattern is consistent with the evocative gene–environment processes described earlier (Klahr et al., 2013; Reiss et al., 2022), suggesting that adolescent‐driven dynamics, rather than direct environmental effects, partly explain the negative link between psychological control and self‐esteem.

### Longitudinal effects of parenting on self‐esteem

Within the ACE‐beta parameterization, perceived maternal warmth and psychological control at age 13 remained prospectively associated with self‐esteem 2 years later after accounting for covariance assigned to common genetic and shared environmental pathways and for age‐13 self‐esteem. The two‐year lag establishes temporal ordering, while adjustment for prior self‐esteem shows that these associations predict age‐15 self‐esteem over and above adolescents' measured level of self‐esteem at age 13. Together, these features strengthen the interpretation of the estimates as prospective associations and reduce the likelihood that they simply reflect temporary reporting tendencies at one assessment or pre‐existing differences in measured self‐esteem.

The positive association for maternal warmth is consistent with research linking warm and supportive parenting to more positive self‐evaluations across childhood and adolescence (Brummelman et al., 2015; Khaleque, 2013). It is also consistent with SDT, which emphasizes the importance of relatedness and autonomy support for healthy psychological development (Ryan & Deci, 2000). Maternal warmth may provide adolescents with repeated experiences of acceptance, responsiveness, and emotional support that reinforce their sense of being valued and worthy. Warm parenting may also support adolescents' growing autonomy while maintaining a secure and supportive parent–child relationship, thereby providing a relational context conducive to positive self‐esteem.

Conversely, higher perceived maternal psychological control at age 13 was prospectively associated with lower self‐esteem at age 15. This pattern is consistent with SDT, which proposes that controlling interpersonal environments can undermine autonomy and competence by restricting individuals' sense of agency (Ryan & Deci, 2000). Controlling parenting may communicate disapproval, constrain adolescents' opportunities to express independent views, or make parental approval appear contingent on compliance, potentially undermining adolescents' developing sense of autonomy and self‐worth.

However, the beta coefficients do not uniquely identify directional effects. Because the ACE‐beta model fixes the cross‐trait nonshared environmental path to zero, it cannot distinguish a directional influence of perceived parenting from twin‐specific experiences, perceptions, response tendencies, or residual measurement processes that contribute to both perceived parenting and later self‐esteem. The significant estimates in the bivariate ACE models indicate that this alternative representation is empirically plausible. The findings are therefore consistent with a directional contribution of perceived maternal parenting to subsequent self‐esteem, but they do not exclude time‐varying nonshared common causes.

### Implications

The prospective associations identify perceived maternal parenting as a meaningful developmental context for adolescent self‐esteem. Maternal warmth was associated with higher later self‐esteem, whereas psychological control was associated with lower later self‐esteem, even after accounting for covariance assigned to common genetic and shared environmental pathways and for prior self‐esteem. These patterns are consistent with the view that everyday parent–adolescent interactions characterized by acceptance, responsiveness, positive communication, and respect for autonomy may support adolescents' sense of worth, whereas controlling interactions may undermine autonomy and positive self‐evaluation.

The findings also highlight the importance of distinguishing shared from nonshared sources of covariance. Shared environmental overlap may reflect broader contexts that make co‐twins similar, such as the general emotional and relational climate of the family. Adolescents within the same family may also differ in their parenting experiences, perceptions, and response tendencies, and these differences may covary with self‐esteem. Because the nonshared environmental component includes residual measurement and factor‐score estimation error, however, the observed nonshared covariance should not be attributed solely to differential parenting experiences or interpreted as evidence of a causal pathway.

Although the present models do not establish that changing parenting will necessarily cause corresponding changes in self‐esteem, the prospective findings, considered alongside the broader longitudinal and intervention literature, support parental warmth and autonomy‐respecting practices as plausible and testable targets for promoting adolescent self‐worth. Parenting‐focused programs that strengthen warmth, responsiveness, positive communication, and autonomy support, while reducing psychologically controlling practices, provide an important context in which these implications can be tested. Intervention studies are needed to determine whether changing these practices produces corresponding improvements in adolescent self‐esteem.

### Limitations and future research directions

Several limitations should be considered. First, the classical twin data cannot simultaneously identify the cross‐trait nonshared environmental path (e_21_) and the directional phenotypic path (β_21_). The beta estimates therefore quantify temporally ordered associations under the identifying constraint e_21_ = 0 and should not be interpreted as definitive causal effects. Repeated measures of both parenting and self‐esteem within genetically informed cross‐lagged or transactional models are needed to evaluate directionality, reciprocity, and time‐varying nonshared common causes more directly.

Second, because the ACE models treated estimated factor scores as observed variables, they did not explicitly separate substantive nonshared environmental variance from residual measurement and factor‐score estimation error. The E estimates should therefore not be interpreted as pure measures of individual‐specific environmental influence, and comparisons with estimates based on raw composites or fully latent twin models should be made cautiously.

Third, the TwinLife “Psychological Control” composite was brief and conceptually heterogeneous, with items spanning punitive discipline, emotion‐based disapproval, and restriction on questioning parental authority. The findings should therefore be interpreted as pertaining to a broad indicator of perceived maternal control and should not be generalized to the full construct of psychological control without replication using a more comprehensive and differentiated measure.

Fourth, ACE models do not fully distinguish different forms of gene–environment correlation. It remains unclear whether genetic covariance reflects child‐driven processes, parent‐driven processes, or both. Extended twin, adoption, or children‐of‐twins designs would help clarify these mechanisms. The present analyses also focused on parenting‐to‐self‐esteem paths; repeated‐measures transactional models are needed to examine reciprocal associations more directly (Kerr et al., 2012; Krauss et al., 2020).

Finally, future research should examine moderation by sex and include paternal parenting. Adolescents may differ in how they interpret or respond to parental warmth and control, and maternal and paternal practices may show distinct genetic and environmental pathways (Cookston & Finlay, 2006; Neiderhiser et al., 2007).

## CONCLUSION

This study advances understanding of the genetic and environmental processes underlying associations between perceived maternal parenting and adolescent self‐esteem. The variance decompositions showed developmentally varying contributions of genetic, shared environmental, and nonshared environmental factors, and the bivariate models identified distinct patterns of overlap for warmth and psychological control. Within the ACE‐beta parameterization, maternal warmth and psychological control at age 13 were prospectively associated with higher and lower self‐esteem, respectively, at age 15 after adjustment for prior self‐esteem and covariates. These estimates are conditional on the ACE‐beta model's identifying constraint and do not uniquely establish directional or causal effects. Considered alongside the broader literature, the findings support warm and autonomy‐respecting parenting as plausible targets for promoting adolescent self‐worth and underscore the need for repeated‐measures genetically informed designs to test directionality more directly.

## AUTHOR CONTRIBUTIONS

**Jasmine Lim:** Conceptualization; methodology; writing – original draft; formal analysis. **Jiesi Guo:** Conceptualization; methodology; validation; supervision; writing – review and editing. **Christoph Klatzka:** Conceptualization; methodology; writing – review and editing. **Ziwen Teuber:** Conceptualization; writing – review and editing.

## FUNDING STATEMENT

This work was supported by a grant from the Australian Research Council awarded to Jiesi Guo (DE230100300).

## CONFLICT OF INTEREST STATEMENT

The authors declare no conflicts of interest.

## ETHICS STATEMENT

Ethical approval for the TwinLife study was granted by the German Psychological Society (Protocol Number: RR 11.2009).

## CONSENT STATEMENT

Informed consent was obtained for all participants in this study. For the adolescent cohort, written informed consent (parental permission) was obtained from their parents or legal guardians. Additionally, all adolescent participants were provided with age‐appropriate information about the study's aims and procedures, and their voluntary assent was obtained prior to data collection. The study protocol and consent procedures were approved by the Ethical Committee of the German Psychological Society (DGPs; Protocol Number RR 11.2009).

## Data Availability

The data that support the findings of this study are available from the TwinLife project via the GESIS Data Archive. Restrictions apply to the availability of these data, which were used under license for the current study. Data are available at https://www.twin‐life.de with the permission of the TwinLife consortium.
